# Major discrepancy between clinical diagnosis of death and anatomopathological findings in adolescents with chronic diseases during 18-years

**DOI:** 10.1016/j.clinsp.2023.100184

**Published:** 2023-03-25

**Authors:** Maira P. Ribeiro, Amaro N. Duarte-Neto, Marisa Dolhnikoff, Livia Lindoso, Benito Lourenço, Heloisa H. Marques, Maria F.B. Pereira, Lilian M. Cristofani, Vicente Odone-Filho, Lucia M.A. Campos, Adriana M.E. Sallum, Magda Carneiro-Sampaio, Artur F. Delgado, Werther B. Carvalho, Thais Mauad, Clovis A. Silva

**Affiliations:** a Child and Adolescent Institute, Hospital das Clinicas, Faculdade de Medicina, Universidade de Sao Paulo (HCFMUSP), São Paulo, SP, Brazil; b Patology Department, Faculdade de Medicina, Universidade de São Paulo (FMUSP), São Paulo, SP, Brazil

**Keywords:** Autopsy, Death, Adolescent, Chronic diseases, Pneumonia, Yeast

## Abstract

**Objectives:**

To evaluate the inconsistency between clinical diagnosis of death and autopsy findings in adolescents with chronic diseases.

**Methods:**

A cross-sectional study including a sample of adolescents’ autopsies who died in a pediatric and adolescent tertiary hospital over 18 consecutive years. During this period, there were n = 2912 deaths, and n = 581/2912(20%) occurred in adolescents. Of these, n = 85/581(15%) underwent autopsies and were analyzed. Further results were divided into two groups: Goldman classes I or II (high disagreement between main clinical diagnosis of death and anatomopathological findings, n = 26) and Goldman classes III, IV or V (low or no disagreement between these two parameters, n = 59).

**Results:**

Median age at death (13.5 [10‒19] vs. 13 [10‒19] years, p = 0.495) and disease duration (22 [0‒164] vs. 20 [0‒200] months, p = 0.931), and frequencies for males (58% vs. 44%, p = 0.247) were similar between class I/II vs. class III/IV/V. The frequency of pneumonia (73% vs. 48%, p = 0.029), pulmonary abscess (12% vs. 0%, p = 0.026), as well as isolation of yeast (27% vs. 5%, p = 0.008), and virus (15% vs. 2%, p = 0.029) identified in the autopsy, were significantly higher in adolescents with Goldman class I/II compared to those with Goldman class III/IV/V. In contrast, cerebral edema was significantly lower in adolescents of the first group (4% vs. 25%, p = 0.018).

**Conclusion:**

This study showed that 30% of the adolescents with chronic diseases had major discrepancies between clinical diagnosis of death and autopsy findings. Pneumonia, pulmonary abscess, as well as isolation of yeast and virus were more frequently identified at autopsy findings in the groups with major discrepancies.

## Highlights

• Evaluation of disagreement between clinical diagnosis of death and autopsy findings in adolescents with chronic diseases.
• 30% of the adolescents with chronic diseases had major discrepancies between clinical diagnosis of death and autopsy findings, mainly caused by infections.
• Pneumonia, pulmonary abscess, as well as isolation of yeast and virus were identified at autopsy findings in the groups with major discrepancies.

## Introduction

The incidence and prevalence of pediatric chronic conditions have increased in most developed and developing countries over the last few years.^1^, ^2^, ^3^, ^4^ Chronic conditions in adolescents may affect growth, puberty, and the maturation of biological systems through chronic inflammation, glucocorticoid, and sub-optimal nutrition, with higher morbidity and mortality rates. [4]

Mortality in adolescents has usually been reported on specific chronic diseases, such as neurological,[5] rheumatological,^6^, ^7^, ^8^ oncological,[8,9] as well as studies including simultaneous analyses of healthy subjects, acute and chronic conditions in both children, adolescents and adults populations.[10,11] We recently identified 20% of deaths in adolescents and young adults with chronic diseases followed in a large academic hospital.[4] In this report, information was only obtained through the death and autopsy certificates, and uniform and systematized analyzes were not performed for all autopsies by the same pathologists.[4]

In fact, an autopsy is considered the gold standard technique and can provide relevant information about the accuracy of diagnosis of death, pediatric patient management, and assessment of treatment efficacy.^9^, ^10^, ^11^, ^12^, ^13^, ^14^, ^15^, ^16^, ^17^, ^18^, ^19^ The main clinical diagnosis of death can be compared with the autopsy findings using the Goldman classification criteria, with five distinct classes.[11,15] These can be further classified into two groups: classes I or II with major discrepancy (high disagreement between the main clinical diagnosis of death and autopsy findings)[14,16, 17, 18, 19] and classes III, IV or V (low or no disagreement between the main clinical diagnosis of death and autopsy). To our knowledge, there is no study that evaluated autopsy findings in a particular population of adolescents with chronic diseases.

Thus, the objective of the present study was to evaluate the disagreement between the clinical diagnosis of death and autopsy findings in adolescents with chronic diseases. Comparisons between demographic data, hospital admissions, supportive measures, and autopsy findings in adolescents with Goldman classes I/II versus classes III/IV/V were also performed.

## Methods

A cross-sectional and retrospective study included a sample of autopsies of patients who died in a university and tertiary hospital in São Paulo city, Brazil. This study was retrospective and approved by the Local Ethics Committee (process n° 59062916.20000.0068) of our hospital, with 207 beds, and did not require an informed consent form.

The inclusion criteria were adolescents diagnosed with a pediatric chronic condition, aged between 10‒19 years and 11 months, who died and performed autopsy during a period of 18 consecutive years (2000‒2017). Exclusion criteria were medical records with insufficient information, insufficient information from the autopsy data, and patients who died from suspected or confirmed external causes, as the post-mortem exam in these cases is performed by the Medical Forensic Service.

The autopsies were requested by the assistant pediatrician when there was uncertainty about the immediate and/or underlying cause of death. All the autopsies were performed at the Pathology Department of our university hospital after written consent was signed by the next-of-kin, and after 8 hours of death. The corpses were examined following the Rokitansky (exam of organ-by-organ) or Letulle (dissection en masse) techniques, and samples from all organs were collected, and then examined by optic microscopy, through Hematoxilin-Eosin stain, as well as special stains when necessary. The autopsies were analyzed under well-established concepts in the medical literature.[20,21] The final autopsy report included both macroscopic and microscopic findings, and they were revised retrospectively for this study.

The following variables were systematically evaluated in medical records, death certificates, and autopsies:

1. Demographic data: age at death, sex, origin and disease duration.
2. Characteristics of hospital admission: the period of last hospitalization, number of previous hospitalizations, death in the Pediatric Intensive Care Unit (PICU), and death in the Emergency Room (ER).
3. Supportive measures during hospital admission: pediatric palliative care, surgical procedures, renal replacement therapy, antibiotics, and antifungals therapies, vasoactive drugs, albumin and blood products transfusion, invasive respiratory support, central venous catheterization, and “do-not-resuscitate order”.[4]
4. Chronic conditions: Preexisting chronic condition was defined according to the duration of signs and/or symptoms (more than three months) and the diagnosis established by the physician's scientific knowledge, accurate methods, or instruments according to specific diagnostic criteria for pediatric chronic conditions.^2^, ^3^, ^4^ The following chronic diseases were assessed: neoplasms (leukemia, lymphoma, osteosarcoma, and other cancers), obesity and overweight, cardiopulmonary diseases (heart failure, cystic fibrosis, congenital heart disease, myocarditis, and chronic pericarditis), hematological (chronic anemia, thrombosis, autoimmune hemolytic anemia, immune thrombocytopenia, sickle cell anemia, coagulation disorders), neurological (epilepsy, chronic non-progressive encephalopathy, hydrocephalus, West syndrome), renal (glomerulonephritis, chronic kidney disease, tubulopathies, nephrotic syndrome), genetic diseases (Turner, Down and Noonan syndromes), autoimmune diseases (type 1 diabetes mellitus, juvenile systemic lupus erythematosus, juvenile idiopathic arthritis, celiac disease, and inflammatory bowel disease), liver diseases (biliary atresia, liver transplantation and other liver diseases) and Acquired Immunodeficiency Syndrome (AIDS).
5. Causes of death described in the death certificate: immediate cause of death (final condition or injury resulting in death) and underlying cause of death (disease or condition that initiated the events resulting in death). All information in the death certificate such as intermediate chain and associated pathologies were categorized on the final clinical diagnosis, and they were considered when analyzing adequacy between autopsy findings and causes of death.
6. Autopsy data: Autopsies were systematically reviewed by two pathologists and included the following systematic macroscopic and microscopic evaluations of organs and systems, such as: cerebral (meningoencephalitis, cerebral hemorrhage, cerebral infarction, cerebral herniation, cerebral edema, ischemia of parenchyma); cardiac (cardiomegaly, left ventricular ischemia, pericardial effusion, myocarditis, endocarditis, pericarditis, myocardiosclerosis); vascular (atherosclerosis, aneurysm, aortic dissection, vasculitis); pulmonary (pneumonia, pleural effusion, pneumothorax, hemothorax, pulmonary abscess, pulmonary hemorrhage, pulmonary thromboembolism, pulmonary edema, pulmonary infarction, bronco aspiration, tracheitis, mediastinal mass); hepatic and biliary (ascites, hepatitis, liver abscess, cirrhosis, biliary obstruction); pancreatic (pancreatitis); renal/urogenital (pyelonephritis, chronic nephropathy, urinary tract obstruction, renal infarction, genital organ alteration); spleen (spleen abscess, spleen necrosis, splenitis); gastrointestinal (esophageal changes, hemorrhagic gastritis, gastric ulcer, colitis, gastrointestinal bleeding, peritonitis, hemoperitoneum, intestinal obstruction, intestinal perforation); other alterations (muscle alterations, joint alterations, skin alterations, genetic alterations, malformations); tumor histogenesis (acute myeloid leukemia and type A and B acute lymphoid leukemia); tumor site (lymphohematopoietic, central nervous system, head and neck, mediastinal, cardiac, pulmonary, hepatic, pancreatic, biliary, gastrointestinal, spleen, renal, pelvic, bone and skin); and microorganism (Gram positive cocci, Gram negative cocci, yeasts, hyphomycetes, protozoa and virus). The confirmation of the microbiological diagnosis was carried out according to the pathological findings observed in the tissues and obtained during the autopsy, using special stains and immunohistochemistry. The Grocott and Ziehl-Neelsen stains were used when fungal or mycobacterial infections were suspected, respectively, and specific monoclonal antibodies to detect viral antigens were used when the viral cytopathic effect was observed in tissues.
7. Goldman classification criteria: For each adolescent patient, the main clinical diagnoses of death were compared with the autopsy findings, by a pediatrician (M.P.R.) and pathologist (A.N.D.N.), using the Goldman criteria, which included five different classes: I to V. According to these criteria, the discrepancies were classified as follows:[11,15]

Class I ‒ Missed major diagnosis with probable impact on survival and that accurate diagnosis would have changed management.
Class II ‒ Missed major diagnosis with no probable impact on survival and that accurate diagnosis would not have changed management.
Class III ‒ Missed minor diagnosis related to terminal illness, but not related to the death cause.
Class IV ‒ Other minor diagnoses ceased to be recognized.
Class V ‒ Absolute agreement between clinical diagnosis of death and autopsy findings.

Further results of the autopsies were divided into two groups: classes I or II (high disagreement between the main clinical diagnosis of death and autopsy findings) and classes III, IV or V (low or no disagreement between the main clinical diagnosis of death and the autopsy findings).

### Statistical analysis

Statistical Package for Social Sciences for Windows 24.0 (IBM Corp., Armonk, NY, USA) was used to perform all statistical analyses. Data were described as median (range) for continuous with non-normal distribution or mean ± Standard Deviation (SD) for continuous with normal distribution variables and a number (frequency) for categorical variables. Scores that had non-normal and normal distributions were compared by the Mann-Whitney test and t-test, respectively. Differences in categorical variables were evaluated according to Fisher's exact test or Pearson Chi-Square test. A p-value < 0.05 was considered statistically significant.

## Results

During this period, there were n = 2912 deaths in all age groups (0 to 19 years and 11 months). Of these deaths, n = 581/2912 (20%) occurred in adolescents. Over 18 consecutive years, autopsies were performed on n = 85/581 (15%) adolescents with chronic conditions and were analyzed in this study.

The most common clinical diagnoses of the primary underlying conditions evidenced in both groups were: neoplasia (n = 6 [23%] vs. n = 21 [36%]), liver diseases/liver transplantation (n = 5 [19%] vs. n = 9 [15%]), juvenile systemic lupus erythematosus (n = 5 [19%] vs. n = 5 [8%]), and AIDS (n = 3 [11.5%] vs. n = 4 [7%]). Of these cases, the autopsies with Goldman class I of discrepancy were n = 20/85 (24%), class II in n = 6/85 (7%), class III in n = 3/85 (3%), class IV in n = 0/85 (0%) and class V in n = 56/85 (66%). The chronic conditions in 85 adolescent and young adult patients comparing results of autopsies according to Goldman classes during an 18 years period were similar in classes I/II (high disagreement, n = 26) compared to those in classes III/IV/V (low/no disagreement, n = 59) (p = 0.553).

Table 1 shows demographic data and characteristics of death in 85 adolescent and young adult patients with chronic diseases comparing results of autopsies according to high disagreement versus low/no disagreement. Median age (13.5 [10‒19] vs. 13 [10‒19]) years, p = 0.495) and male sex (58% vs. 44%, p = 0.247) were similar in both groups. No differences were evidenced in the other demographic data, hospital admissions, and supportive measures at PICU or ER (p > 0.05), (Table 1).

**Table 1:** Demographic data and characteristics of death in 85 adolescent and young adult patients with chronic diseases comparing results of autopsies according to Goldman classes during 18 years period: classes I/II (high disagreement) versus classes III/IV/V (low/no disagreement).

Variables	Class I/II (n = 26)	Class III/IV/V (n = 59)	p
**Demographic data**			
Age at death, years	13.5 (10‒19)	13 (10‒19)	0.495
Male sex	15 (58)	26 (44)	0.247
Residents out of the state of SP	3 (12)	8 (14)	1.000
Disease duration, months	22 (0‒164)	20 (0‒200)	0.931
**Hospital admissions at PICU or ER**			
Period of last hospitalization, days	23 (0‒102)	9 (0‒99)	0.563
Number of previous hospitalizations	3.5 (1‒19)	3 (1‒32)	0.448
Death at PICU	18 (69)	44 (75)	0.609
Death at ER	6 (23)	7 (12)	0.204
**Supportive measures at PICU or ER**			
Pediatric palliative care	0 (0)	3 (5)	0.550
Surgical procedures	8 (31)	20 (34)	1.000
Renal replacement therapy	7 (27)	14 (24)	0.789
Antibiotics therapy	23 (88)	55 (93)	0.670
Antifungal therapy	13 (50)	36 (61)	0.344
Vasoactive drugs	25 (96)	49 (83)	0.160
Albumin transfusion	10 (38)	22 (37)	1.000
Blood products transfusion	18 (69)	49 (83)	0.162
Invasive respiratory support	23 (88)	53 (90)	1,000
Central venous catheterization	0 (0)	0 (0)	NA
“Do-not-resuscitate order”	5 (19)	7 (12)	0.500

Results are presented in n (%), median (minimum-maximum); Class I, Missed major diagnosis with probable impact on survival and that accurate diagnosis would have changed management; Class II, Missed major diagnosis with no probable impact on survival and that accurate diagnosis would not have changed management; PICU, Pediatric Intensive Care Unit; ER, Emergency Room; NA, Not Applicable; SP, São Paulo.

According to the death certificate data, the immediate cause of death in 85 adolescent and young adult patients comparing results of autopsies according to Goldman classes were similar in classes I/II (high disagreement, n = 26) compared to those in classes III/IV/V (low/no disagreement, n = 59) (p = 0.435). The main immediate cause of death evidenced in both groups were: pneumonia (n = 11 [42%] vs. n = 15 [25%]), pulmonary hemorrhage (n = 1 [4%] vs. n = 5 [8%]), hemorrhagic shock (n = 1 [4%] vs. n = 4 [7%]), and invasive aspergillosis (n = 4 [15%] vs. n = 2 [3%]). The underlying cause of death was also similar in classes I/II compared to those with classes III/IV/V, and the main underlying cause of death evidenced in both groups was: neoplasia (n = 3 [11%] vs. n = 22 [37%]), juvenile systemic lupus erythematosus (n = 5 [19%] vs. n = 5 [8%]), liver cirrhosis (n = 1 [4%] vs. n = 6 [10%]), and AIDS (n = 3 [11%] vs. n = 4 [7%]).

Tables 2 and 3 show autopsy findings in 85 adolescent and young adult patients with chronic diseases according to Goldman classes during an 18 years period: classes I/II (high disagreement, n = 26) versus classes III/IV/V (low/no disagreement, n = 59). The frequencies of pneumonia (73% vs. 48%, p = 0.029), pulmonary abscess (12% vs. 0%, p = 0.026), as well as isolation of yeast (27% vs. 5%, p = 0.008), and virus (15% vs. 2%, p = 0.029) identified in the autopsy were significantly higher in adolescents with Goldman class I/II compared to those with Goldman class III/IV/V (Tables 2 and 3). In contrast, cerebral edema was significantly lower in adolescents with Goldman class I/II compared to those with Goldman class III/IV/V autopsies (4% vs. 25%, p = 0.018) (Table 2).

**Table 2:** Autopsy findings in 85 adolescent and young adult patients with chronic diseases according to Goldman classes during 18 years period: classes I/II (high disagreement) versus classes III/IV/V (low/no disagreement).

Autopsy findings	Class I/II (n = 26)	Class III/IV/V (n = 59)	p
**Cerebral involvement**	12 (46)	30 (51)	0.690
Meningoencephalitis	7 (27)	7 (12)	0.114
Cerebral hemorrhage	7 (27)	17 (29)	1.000
Cerebral herniation	2 (8)	3 (5)	0.639
Cerebral edema	1 (4)	15 (25)	**0.018**
Parenchyma ischemia	0 (0)	1 (2)	1.000
**Heart involvement**	10 (38)	27 (46)	0.532
Cardiomegaly	2 (8)	5 (8)	1.000
Left ventricle hypertrophy	0 (0)	1 (2)	1.000
Pericardial effusion	4 (15)	19 (32)	0.108
Myocarditis	6 (23)	5 (8)	0.084
Pericarditis	1 (4)	1 (2)	0.521
Myocardiosclerosis	1 (4)	1 (2)	0.521
**Pulmonary involvement**	23 (88)	56 (95)	0.364
Pneumonia	19 (73)	28 (47)	**0.029**
Pleural effusion	11 (42)	29 (49)	0.560
Pneumothorax	1 (4)	0 (0)	0.306
Hemothorax	1 (4)	1 (2)	0.521
Pulmonary abscess	3 (12)	0 (0)	**0.026**
Pulmonary hemorrhage	10 (38)	28 (47)	0.486
Pulmonary thromboembolism	2 (8)	2 (3)	0.583
Pulmonary edema	7 (27)	21 (36)	0,433
Pulmonary infarction	0 (0)	3 (5)	0.550
Pulmonary bronco aspiration	0 (0)	2 (3)	1.000
Tracheitis	0 (0)	2 (3)	1.000
Mediastinal mass	1 (4)	0 (0)	0.306
**Hepatic and biliary involvements**	13 (50)	29 (49)	1.000
Ascites	9 (35)	24 (41)	0.808
Hepatitis	7 (27)	11 (19)	0.389
Hepatic abscess	1 (4)	1 (2)	0,521
Cirrhosis	1 (4)	3 (5)	1.000
**Pancreas involvement**	6 (23)	14 (24)	0.948
Pancreatitis	6 (23)	14 (24)	0.948
**Renal involvement**	10 (38)	12 (20)	0.079
Pyelonephritis	5 (19)	5 (8)	0.155
Chronic nephropathy	5 (19)	6 (10)	0.299
Renal infarction	0 (0)	1 (2)	1.000
Genital organ alteration	2 (8)	1 (2)	0.220
**Spleen involvement**	11 (42)	26 (44)	0.880
Spleen necrosis	2 (8)	5 (8)	1.000
Splenitis	10 (38)	23 (39)	0.930

Results are presented in n (%); Class I, Missed major diagnosis with probable impact on survival and that accurate diagnosis would have changed management; Class II, Missed major diagnosis with no probable impact on survival and that accurate diagnosis would not have changed management; NA, Not Applicable.

**Table 3:** Another autopsy findings in 85 adolescent and young adult patients with chronic diseases according to Goldman classes during 18 years period: classes I/II (high disagreement) versus classes III/IV/V (low/no disagreement).

Autopsy findings	Class I/II (n = 26)	Class III/IV/V (n = 59)	p
**Gastrointestinal involvement**	17 (65)	34 (58)	0.501
Esophagus alterations	6 (23)	12 (20)	0.779
Hemorrhagic gastritis	7 (27)	18 (31)	0.738
Gastric ulcers	5 (19)	4 (7)	0.124
Colitis	8 (31)	12 (20)	0.482
Digestive hemorrhage	6 (23)	13 (22)	0.915
Peritonitis	2 (8)	2 (3)	0.583
Hemoperitoneum	1 (4)	0 (0)	0.306
Intestinal obstruction	0 (0)	2 (3)	1.000
Intestinal perforation	0 (0)	1 (2)	1.000
**Other abnormalities**	13 (50)	35 (59)	0.424
Muscles abnormalities	0 (0)	2 (3)	1.000
Joint abnormalities	2 (8)	2 (3)	0.583
Skin abnormalities	9 (35)	27 (46)	0.338
Genetic abnormalities	2 (8)	6 (10)	1.000
Malformations	0 (0)	5 (8)	0.317
**Tumors**	8 (31)	21 (36)	0.805
Tumor primary histogenesis			
Acute myeloid leukemia	1 (4)	4 (7)	1.000
Acute lymphoblastic leukemia A	0 (0)	3 (5)	0.550
Acute lymphoblastic leukemia B	1 (4)	2 (3)	1.000
Others	6 (23)	12 (20)	0.779
**Tumor primary site**			
Lymphohematopoietic	3 (12)	16 (27)	0.159
Armpit	0 (0)	1 (2)	1.000
Inferior limbs	0 (0)	3 (5)	0.550
Central nervous system tumor	1 (4)	0 (0)	0.306
Retro orbital	1 (4)	0 (0)	0.306
Skull	1 (4)	0 (0)	0.306
Mediastinum	1 (4)	0 (0)	0.306
Intestine	1 (4)	0 (0)	0.306
Colon	0 (0)	1 (2)	1.000
**Localization of tumor**			
Central nervous system	2 (8)	3 (5)	0.639
Head and neck	1 (4)	2 (3)	1.000
Mediastinal	1 (4)	4 (7)	1.000
Cardiac	0 (0)	2 (3)	1.000
Pulmonary	2 (8)	4 (7)	1,000
Hepatic	2 (8)	8 (14)	0.717
Pancreatic	0 (0)	1 (2)	1.000
Bile ducts	0 (0)	1 (2)	1.000
Intestinal	0 (0)	4 (7)	0.308
Spleen	2 (8)	9 (15)	0.491
Kidney	0 (0)	4 (7)	0.308
Pelvis	0 (0)	1 (2)	1.000
Bone	3 (12)	7 (12)	1.000
**Microorganism**			
Gram positive cocci	2 (8)	0 (0)	0.091
Gram negative cocci	1 (4)	0 (0)	0.306
Yeasts	7 (27)	3 (5)	**0.008**
Hyphomycetes	3 (12)	1 (2)	0.083
Protozoa	1 (4)	0 (0)	0.306
Virus	4 (15)	1 (2)	**0.029**

Results are presented in n (%); Class I, Missed major diagnosis with probable impact on survival and that accurate diagnosis would have changed management; Class II, Missed major diagnosis with no probable impact on survival and that accurate diagnosis would not have changed management; NA, Not Applicable.

Table 4 includes clinical and anatomopathological diagnoses according to autopsies in adolescent and young adult patients with chronic diseases and major discordance in autopsies according to Goldman classes I or II (n = 26/85 [31%]). The main cause of high disagreement between the main final clinical diagnosis of death and autopsy findings (Goldman class I or II) was infection identified at autopsy findings, which was evidenced in n = 19/26 (73%) patients. Fifteen of 26 (58%) patients with Goldman classes I or II received the final clinical diagnosis of sepsis/septic shock without a primary defined source of infection and 12/26 (46%) of them had a definitive diagnosis of pneumonia, aspiration pneumonia, and/or aspergillosis through necropsy. All of these 16/26 (61%) patients with Goldman classes I or II had the final clinical diagnosis of sepsis/septic shock without a primary defined source of infection, based on a combination of signs, symptoms that indicate sepsis after careful analysis of retrospective clinical, laboratory and radiological data from each patient's medical record. Pneumonia was the final autopsy diagnosis in 13/26 (50%) patients with Goldman classes I or II and had not been previously diagnosed (Table 4).

**Table 4:** Clinical and anatomopathological diagnoses according to autopsies in adolescent and young adult patients with chronic diseases and major discordance in autopsies according to Goldman classes I or II.

Patient	Age at death	Sex	Final clinical diagnosis (other relevant information of clinical history on medical records)	Final autopsy diagnosis^a^	Goldman class
1	11	M	**Pneumonia** (JIA, fever, vomiting, seizure)	Diffuse hemophagocytosis	I
2	12	F	**Pneumonia and sepsis/septic shock** (JIA)	Pneumonia/cytomegaloviruses	I
3	13	M	**Bacterial peritonitis and sepsis/septic shock** (hepatic cirrhosis)	Disseminated candidiasis	I
4	11	F	**Sepsis/septic shock without a primary defined source of infection** (medulloblastoma, cerebral herniation)	Pneumonia	II
5	11	M	**Severe drug reaction** (Stevens-Johnson syndrome after penicillin allergy)	Pneumonia	I
6	11	M	**Intestinal sub occlusion and sepsis/septic shock without a primary defined source of infection** (PNET, abdominal distension, hematemesis, dehydration)	Hemorrhagic shock by bowel perforation	I
7	16	F	**Febrile neutropenia** (AML)	Diffuse severe hemorrhage by coagulopathy	I
8	11	F	**Sepsis/septic shock without a primary defined source of infection** (congenital hepatic fibrosis)	Pneumonia due to *Nocardia*	I
9	16	M	**Sepsis/septic shock without a primary defined source of infection** (sclerosis cholangitis after liver transplantation)	Acute pancreatitis	I
10	18	F	**Hypovolemic shock** (cSLE, arterial hypertension)	Disseminated intravascular coagulation	I
11	14	M	**Sepsis/septic shock without a primary defined source of infection** (germ cell tumor, ARDS)	Pneumonia/cytomegaloviruses	II
12	19	F	**Pericarditis and sepsis/septic shock without a primary defined source of infection** (cSLE)	Invasive aspergillosis	I
13	18	M	**Sepsis/septic shock without a primary defined source of infection** (sclerosis cholangitis after liver transplantation, ARDS)	Aspiration pneumonia	I
14	11	F	**Disseminated angiosarcoma and sepsis/septic shock without a primary defined source of infection** (pancytopenia)	Cerebral hemorrhage	I
15	13	F	**Pancytopenia, pneumonia and sepsis/septic shock** (cSLE)	Invasive aspergillosis	II
16	14	M	**Acute respiratory insufficiency** (juvenile dermatomyositis, pulmonary hemosiderosis)	Pneumonia	I
17	14	F	**Liver insufficiency and sepsis/septic shock without a primary defined source of infection** (alpha 1-antitrypsin deficiency)	Invasive aspergillosis	I
18	11	M	**Chronic diarrhea and sepsis/septic shock without a primary defined source of infection** (HIV)	*Cryptococcosis*	I
19	12	M	**Neurotoxoplasmosis and sepsis/septic shock without a primary defined source of infection** (HIV)	Invasive aspergillosis	I
20	12	F	**Sepsis/septic shock without a primary defined source of infection** (ALL, and severe musculoskeletal pain)	Pneumonia/necrotising bacterial laryngitis	II
21	10	M	**Cardiorespiratory arrest** (HIV)	Pneumonia by *Pneumocystis jirovecii*	I
22	17	F	**Sepsis/septic shock without a primary defined source of infection** (cSLE, hypogammaglobinemia, fever, vomiting, headache)	Pneumonia	II
23	15	M	**Sepsis/septic shock without a primary defined source of infection** (epidermolysis bullosa)	Pneumonia/sigmoid volvulus	I
24	16	M	**Sepsis/septic shock without a primary defined source of infection** (cSLE, fever, vomiting, myalgia, seizure)	Pneumonia	II
25	14	M	**Alveolar hemorrhage** (medullary aplasia, fever, bronchoalveolar culture positive for *Elizabethkingia meningoseptica*)	Septic shock due to disseminated candidiasis	I
26	17	M	**Diffuse hemorrhage** (idiopathic thrombocytopenic purpura, fever, hypoxemia, disseminated petechial rash, meningeal signs)	Pneumonia/mediastinal abscess	I

Class I, Missed major diagnosis with probable impact on survival and that accurate diagnosis would have changed management; Class II, Missed major diagnosis with no probable impact on survival and that accurate diagnosis would not have changed management; F, Female; M, Male; JIA, Juvenile Idiopathic Arthritis; PNET, Primitive Neuroectodermal Tumour; AML, Acute Myeloid Leukemia, ALL, Acute Lymphoid Leukemia; cSLE, childhood Systemic Lupus Erythematosus; ARDS, Acute Respiratory Distress Syndrome; HIV, Human Immunodeficiency Virus.

a Final autopsy diagnosis is based on macroscopic and microscopic analyses.

## Discussion

This study showed that 30% of adolescents with chronic diseases had major discrepancies between clinical diagnosis of death and autopsy findings, mainly due to infections. Pneumonia, pulmonary abscess, as well as isolation of yeast and virus were the major findings related to the discrepancies.

The great strength of the present study was the inclusion of a unique adolescent population followed in a large tertiary hospital. Our academic hospital is one of the Brazilian reference centers for pediatric and complex specialties that follows various chronic conditions.[2, 3, 4,22] A strong point observed herein was the long-term period of an autopsy, evaluating 18 consecutive years. Another strength of this study was that the autopsies were performed in the same Pathology Department, with all organs routinely examined, including the characterization of microorganisms. Moreover, all autopsies were revised for this study.

We confirmed previous studies demonstrating that one-third of autopsies had a high disagreement between the main clinical diagnosis of death and anatomopathological findings. In fact, major errors with relevant clinical and pathological discrepancies have been reported from 0.3% to 32% of autopsies.[10,14, 15, 16, 17-19] A systematic review of pediatric studies including diagnostic error showed that major diagnostic discrepancies were observed in approximately 20% of autopsies in PICU and neonatal ICU admissions.[15]

We extended previous observations and showed that infections were the most important misdiagnoses and possibly were not identified during hospitalization, particularly pneumonia and pulmonary abscess. Indeed, infections may be related to higher frequencies of immunosuppressive conditions observed in the present study, particularly neoplasia, chronic liver, rheumatic and infectious diseases. The use of immunosuppressants or chemotherapy drugs and disease activity seem to be contributing factors for infection-related deaths.

Regarding the isolation of microorganisms, approximately one-third of major errors occurred in autopsies of adolescents in whom yeasts were identified, and 15% occurred in patients with viral infections. Fungal and viral infections are relevant for hospitalized patients with neoplasia, chronic liver, rheumatic and infectious diseases.[2, 3, 4,6,8,22, 23, 24, 25] Furthermore, antifungal therapy was used in half of the adolescents with Goldman Classes I or II, and may have influenced the higher frequencies of misdiagnoses of these infections in the groups with major discrepancies.

Moreover, pneumonia has been frequently reported in deceased adolescents with chronic conditions.[4] Misdiagnosed pneumonia and lung abscess were observed herein and contributed to major clinical-pathological discrepancies, as also identified by other series.[26,27] These results reinforce suspicion of systematic pulmonary infections in the PICU and ER, as well as the implementation of accurate diagnostic methods. The high frequency of pneumonia in 50% of our autopsies with Goldman classes I and II may be related to long-term period assessment with difficulty to identify etiological agents (particularly viruses and fungal infections), especially in the first years of analysis.

Of note, the autopsy is a relevant tool to confirm clinical diagnoses; however, autopsy rates have been decreasing in the past few years. The decline in autopsy rates may be related to the high number of patients in this institution with severe chronic diseases and long-term follow-up, increasing confidence of pediatricians in new and accurate diagnostic and imaging methods, and parental refusal based on religious, and ethical beliefs, and unfamiliarity with this technique.[4,17] Therefore, strategies to improve adherence to autopsy in the clinical practice of adolescent critical care require continuous awareness by multidisciplinary and multi-professional teams at pediatric university hospitals. A study showed that the implementation of an educational program in PICU had an increase to more than 50% of the autopsy rate compared to 20%‒30% in previous years.[13]

The present study has limitations due to its retrospective design with possible missing data; it is a one-center study, and this institution is a tertiary university hospital and may not be representative of all hospitals in Brazil and Latin America; potential for selection bias, since only 15% of adolescents who died over the study period underwent autopsy. We also did not evaluate autopsy microbiology, since systematic analyses of molecular diagnosis and molecular biology in anatomopathological fragments are relevant to identify specific infectious agents.[25] Further studies will be necessary to clarify this issue.

In conclusion, high discordance between the main clinical diagnosis of death and the anatomopathological findings occurred in approximately one-third of adolescents with chronic diseases, mainly due to infection identified at autopsy. Pneumonia, lung abscess, yeasts, and virus identification occurred predominantly in autopsies of adolescents with a high degree of disagreement between clinical and pathological diagnosis. These data reinforce the necessity to improve microbiological diagnoses and implement rapid proper antimicrobial therapeutic measures in the clinical management of adolescents with chronic and severe diseases.

## Conflicts of interest

The authors declare no conflicts of interest.
